# Nutritional Status, Refeeding Syndrome and Some Associated Factors of Patients at COVID-19 Hospital in Vietnam

**DOI:** 10.3390/nu15071760

**Published:** 2023-04-04

**Authors:** Linh Thuy Nguyen, Thanh Van Ta, An Tuong Bui, Sy Nam Vo, Ngoc-Lan Thi Nguyen

**Affiliations:** 1 Hanoi Medical University Hospital, Hanoi Medical University, 1st Ton That Tung, Dong Da District, Hanoi 100000, Vietnam; 2 Department of Nutrition and Food Safety, School of Training Preventive Medicine and Public Health, Hanoi Medical University, 1st Ton That Tung, Dong Da District, Hanoi 100000, Vietnam; 3 Center for Biomedical Informatics, Vingroup Big Data Institute, Hai Ba Trung District, Hanoi 100000, Vietnam; 4 College of Engineering and Computer Science, VinUniversity, Gia Lam District, Hanoi 100000, Vietnam

**Keywords:** nutritional status, refeeding syndrome, GLIM, COVID-19

## Abstract

Multisystem inflammatory syndrome is associated with COVID-19 and can result in reduced food intake, increased muscle catabolism, and electrolyte imbalance. Therefore COVID-19 patients are at high risk of being malnourished and of refeeding syndrome. The present study aimed to determine the prevalence and correlates of malnutrition and refeeding syndrome (RS) among COVID-19 patients in Hanoi, Vietnam. This prospective cohort study analyzed data from 1207 patients who were treated at the COVID-19 hospital of Hanoi Medical University (HMUH COVID-19) between September 2021 and March 2022. Nutritional status was evaluated by the Global Leadership Initiative on Malnutrition (GLIM) and laboratory markers. GLIM-defined malnutrition was found in 614 (50.9%) patients. Among those with malnutrition, 380 (31.5%) and 234 (19.4%) had moderate and severe malnutrition, respectively. The prevalence of risk of RS was 346 (28.7%). Those with severe and critical COVID symptoms are more likely to be at risk of RS compared to those with mild or moderate COVID, and having severe and critical COVID-19 infection increased the incidence of RS by 2.47 times, compared to mild and moderate disease. There was an association between levels of COVID-19, older ages, comorbidities, the inability of eating independently, hypoalbuminemia and hyponatremia with malnutrition. The proportion of COVID-19 patients who suffered from malnutrition was high. These results underscore the importance of early nutritional screening and assessment in COVID-19 patients, especially those with severe and critical infection.

## 1. Introduction

The COVID-19 pandemic is a global pandemic that caused by severe acute respiratory syndrome coronavirus 2 (SARS-CoV-2). The SARS-CoV-2 virus uses enzyme receptor 2 as a receptor to enter lymphocytes, monocytes, alveolar cells, esophageal epithelial cells, enterocytes and the colon [1]. As of June 2022, the pandemic of coronavirus disease 2019 (COVID-19) has caused more than 540 million cases and 6.3 million deaths [2]. The mortality rate of COVID-19 varies by country, race, socioeconomic status, age and comorbidities as well as nutritional status. Malnutrition in COVID-19 patients is caused by an elevated catabolic state, due to immune-driven inflammatory responses, leading to ‘cytokine storm’ [3]. Nutrition requirement is increased in COVID-19 patients due to increased stress on multiple organs, such as fibrotic damage to the lung parenchyma and the spread of bacteria toxication which necessitates the use of renal replacement therapy. Nutritional intake among COVID-19 patients is generally inadequate due to the presence of clinical symptoms (fatigue, dyspnea, fever, confusion, headache, sore throat, chest pain, pneumonia, diarrhea, nausea and vomiting, and loss of taste and smell) and the increase of inflammatory state [4]. The incidence of malnutrition among the suffered patients, especially older adults with severe COVID-19, has been increasing remarkably. This makes nutrition management an important aspect of nutritional care [5]. A number of studies reported the prevalence of malnutrition and showed that poor nutritional status could worsen prognosis, length of stay in hospital, the ability of the ICU admission and mortality. In recent years, the Global Leadership Initiative on Malnutrition (GLIM) has been used more frequently to diagnose malnutrition. In addition, several laboratory markers can also be measured to reflect nutritional status, including vitamins B1, B6, B12, folate, vitamin D (25-hydroxyvitamin D), selenium, and zinc 6. HMUH COVID-19 is one of the largest treatment hospitals for COVID-19 patients in Vietnam. During the outbreak of the pandemic (from November 2021 to February 2022), the hospital received approximately 500 patients per month. We observed that many patients suffered from malnourishment and cachexia despite good overall fitness before admission. The longer the hospital stay, the more severe the disease and the more complex the comorbidities, the more serious malnutrition that COVID-19 patients may experience. Refeeding syndrome (RS) is also a clinical complication frequently seen in critically ill COVID-19 patients. There is limited understanding of RS and its management among Vietnamese medical staffs due in part to a lack of universally accepted, internationally recognized guidelines for the detection and diagnosis of RS. This study aimed to identify the prevalence of malnutrition and RS among COVID-19 patients and assess factors associated with malnutrition and RS. 

## 2. Materials and Methods

### 2.1. Study Design and Subjects

**Figure 1:**
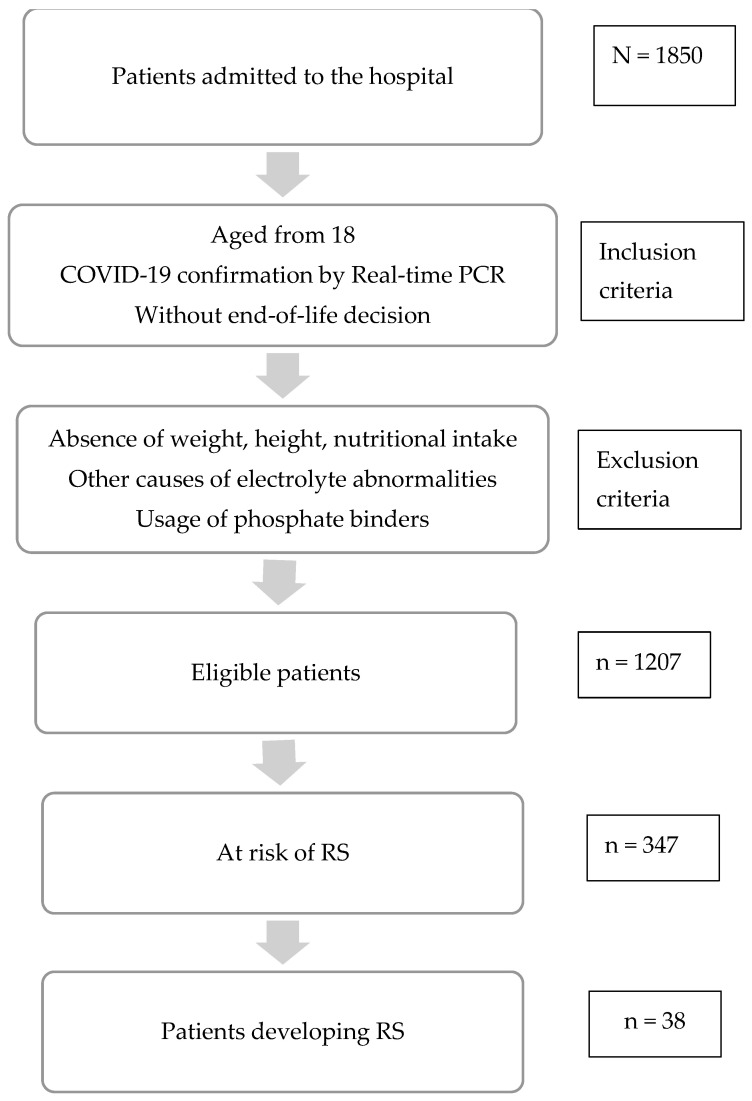
Selection of the study participants.

A prospective cohort study was conducted at COVID-19 hospital, Hoang Mai district, Hanoi, Vietnam, from September 2021 to March 2022 (Figure 1). COVID-19 hospital has 500 beds and is one of the largest front-line hospitals treating COVID-19 patients in Vietnam. The inclusion criteria were: (1) aged from 18, (2) diagnosed with COVID-19 per Realtime-PCR test, (3) hospitalized patients including ICU patients, (4) without end-of-life decisions. Patients with no data on weight, height, nutritional intake, causes of electrolyte disorders such as hyperparathyroidism, or usage of phosphate binders were excluded from our study.

Risk of RS was assessed using the ASPEN guideline. Nourishment was started during the first 48 h after admission to the hospital. After feeding, all the patients were followed within 5 days to note the onset of RS.

### 2.2. Socioeconomic Characteristics and Clinical Information

Information regarding age, gender, comorbidities, severity of COVID-19, oxygen therapy were collected.

### 2.3. Classification of Severity of COVID-19

Severity of COVID-19 infection was classified into four categories: mild, moderate, severe and critical, per the guidelines for diagnosis and treatment of COVID-19 issued together with Decision 4689/QD-BYT dated 6 October 2021 by the Vietnamese Ministry of Health. The clinical determination of each severity category are as follow.

- Mild: Patients had non-specific clinical symptoms such as fever, dry cough, sore throat, stuffy nose, fatigue, headache, muscle aches, loss of taste, smell, or diarrhea., breathing rate < 20 breaths/min and SpO_2_ > 96% when breathing air, normal or minimal damage chest X-ray. The patients are alert and can self-service.
- Moderate: Patient are alert, had signs of pneumonia with shortness of breath, including rapid breathing at 20–25 times/minute, lungs crackles and no signs of severe respiratory distress, and SpO_2_ 94–96% on room air. The patient might have difficulty breathing on exertion such as when walking around the house or going up the stairs. Patient had fast or slow pulse, dry skin, tachycardia, normal blood pressure.
- Severe: Patients had signs of pneumonia accompanied by any of the following: breathing > 25 breaths/minute; severe shortness of breath, contraction of accessory respiratory muscles, or SpO_2_ < 94% when breathing room air. Patients had tachycardia or possibly bradycardia, and BP normal or elevated. Patient might be restless, lethargic, and tired. Patients had chest X-ray and chest CT that showed lesions with lesions more than 50%. Patients had PaO_2_/FiO_2_ 200–300 mmHg, lung ultrasound showing multiple B-lines.
- Critical: Patient had breathing at >30 breaths/minute or <10 breaths/minute, with signs of severe respiratory failure such as labored or irregular breathing. Patient had decreased consciousness or were in coma. Patients had tachycardia or possibly bradycardia, and low blood pressure. Patient had little urine or anuria. Patient had chest X-ray and chest CT showing lesions, with lesions more than 50%. Patient had PaO_2_/FiO_2_ < 200, respiratory acidosis, and blood lactate > 2 mmol/L. Patient had lung ultrasound showing multiple B-lines.

### 2.4. Assessment of Nutritional Status

In this study, included in anthropometric measurements were height, weight, and body mass index (BMI). the Global Leadership Initiative on Malnutrition (GLIM) was used to determine nutritional status based on phenotypic and etiologic criteria. The phenotypic criteria included (i) non-volitional weight loss >5% within the past 6 months or >10% beyond 6 months; (ii) BMI <18.5 kg/m^2^ for age <70 years or <20 kg/m^2^ for age ≥70 years; and (iii) reduced muscle mass. The GLIM criteria suggest that a reduced muscle mass should be diagnosed based on a body composition assessment (e.g., bioelectrical impedance analysis, computed tomography, or dual-energy X-ray absorptiometry). However, as the instruments were not available in COVID-19 hospital, we evaluated muscle mass loss based on clinical examination of medical staffs subjectively. BMI was classified according to the Asia-Pacific classification of BMI, in which BMI of <18.5 kg/m^2^ was considered underweight; 24.9 > BMI ≥ 23 was considered overweight; and BMI ≥ 25 was considered obesity. Reduced food intake or assimilation and evidence of inflammation or chronic disease-related effect were different aspects of etiology. Reduced food intake was evaluated at baseline using quartiles by a dietitian and food assimilation per clinical record. Nutritional status assessment was performed in the first 24–48 h from admission day.

We also tested for several laboratory markers such as serum albumin, prealbumin, sodium, potassium, phosphorus, and triglyceride in first 24–48 h of admission. The normal values for these categories were 35 g/L; 20 mg/dL; 136 mmol/L; 3.4 mmol/L; 0.81 mmol/L; and 1.7 mmol/L, respectively. 

### 2.5. Assessment of Risk of Refeeding Syndrome and Diagnosis of Refeeding Syndrome (RS)

We assessed RS risk according to the American Society of Parenteral and Enteral Nutrition (ASPEN) consensus in 2020 [6].

Risk of refeeding syndrome was determined according to ASPEN 2020 as follow:
Significant risk for RS: 1 out of following criteriaModerate risk for RS: 2 out of following criteria- BMI: <16.0 kg/m^2^- BMI: 16–18.5 kg/m^2^- Weight loss about 10% in 6 months- Weight loss about 5% in 1 month- Severe muscle mass loss, subcutaneous fat loss- Moderate muscle mass loss, subcutaneous fat loss- Caloric intake before admission < 50% energy requirement in 1 week or longer- Caloric intake before admission < 75% energy requirement in 1 week or longer- Moderately or significantly depletion of serum potassium, phosphorus- Minimally depletion of serum potassium, phosphorus

Patients at-risk of RS were follow for 5 days to confirm RS. RS diagnosis was outlined in ASPEN as follows:

- A decrease in any 1, 2, or 3 of serum phosphorus, potassium, and/or magnesium levels by 10–20% (mild RS), 20–30% (moderate RS), or >30% and/or organ dysfunction resulting from a decrease in any of these and/or due to thiamin deficiency (severe RS).
- And occurring within 5 days of reinitiating or substantially increasing energy provision

### 2.6. Data Collection and Analysis

Data was collected by nutritional doctors and dietitians and nurses who directly worked at the ward, then the data was entered into electronic medical records. Data of the study was computerized by the Epidata application version 3.1 and analyzed by the Stata version 13.0 (StataCorp. LP). Descriptive analysis was applied to present information on general characteristics, clinical and biochemical results and nutritional status. To test the differences in characteristics, the ANOVA test and the Kruskal-Wallis test and *t*-test were used for quantitative variables and Chi-square was used for qualitative variables. *p* value < 0.05 was statistically different.

## 3. Results

### 3.1. Characteristics of the Study Participants

General characteristics of the study participants are shown in Table 1. A total of 1207 patients were enrolled in the study. Of these patients, 603 (49.9%) were male and 604 (50.1%) were female. The average age was 58.9 ± 20.7 years. The youngest patient was 18 years old and the oldest patient was 103 years old. 344 (28.5%) patients did not have comorbidities, 586 (48.6%) of all had 1-2 background diseases, and had 277 (22.9%) of patients had more than three comorbidities. More than half of patients were diagnosed with mild COVID-19, 235 (19.5%) of them was moderate, 132 (10.9%) was severe and 204 (16.9%) was in critical condition. In terms of respiratory support, the percentage of patients breathing room air accounted for 749 (62.1%), followed by patients who used masks, then patients using nasal prongs. Patients who used Mechanical ventilation/ ECMO and High Flow Nasal Cannula (HFNC) had a prevalence of 106 (8.8%) and 15 (1.2%) of all patients. The last 15 patients (equivalent to 1.2%) used noninvasive ventilation (NIV).

### 3.2. Nutritional Status among COVID-19 Patients

The overall proportion of malnutrition according to GLIM was 614 (50.9%). Of these, about 380 (31.5%) of patients was diagnosed moderate malnutrition and 234 (19.4%) was severe malnitrition.

**Figure 2:**
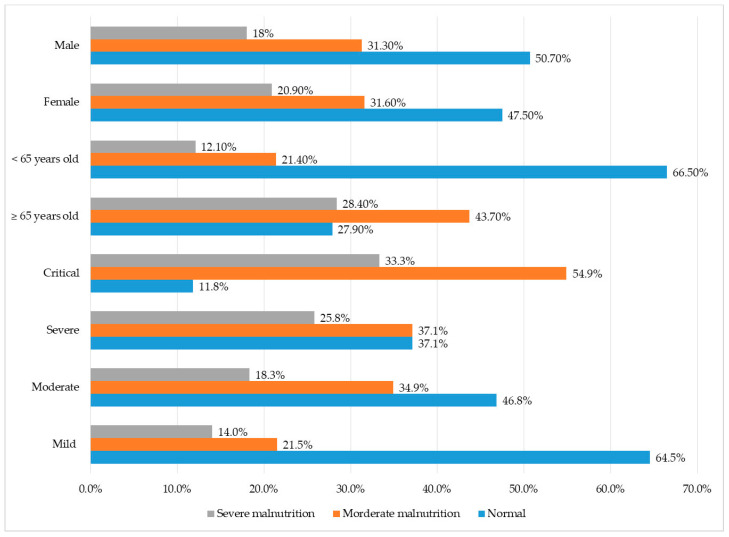
The percentage of malnutrition according to severity of COVID-19, gender and age.

Figure 2 provides that malnutrition occurs at all diagnostic levels. The percentage of moderate and severe malnutrition in severe and critical COVID-19 accounted for the largest figure. In contrast, the proportion of normal nutrition was the highest in mild and moderate COVID-19. There was no difference of the prevalence of malnutrition between two genders. Regarding age groups, the rate of malnutrition among the elderly was much higher than that of people under 65 years of age. The majority of those who are 65 years old or older (72.1%) had moderate or severe malnutrition.

Table 2 indicates that serum albumin decreased paralelly to the severity of COVID-19. the difference was statistically significant. Mean value of sodium and potassium in all levels of the disease were significantly different. but serum sodium was not decreased according to the severity. and potassium climbed following the severity. Even though there were not any statistical difference of mean value of pre-albumin, phosphorus and triglyceride, the tendency of low pre-albumin, hypophosphatemia and hypertriglyceridemia were recorded.

As described in Table 3, patients who were 65 years old or older were significantly more likely to have malnutrition compared to those <65 years old (OR: 5.1 [4–6.6]). Female patients were also more likely than their male counterparts to have malnutrition, although this was not statistically significant (OR: 2.4 [1.8; 3.1]. *p* > 0.05). According to BMI, older population also had a higher risk of malnutrition (OR: 1.6 [1–2.4]). Patients who had severe or critical COVID-19 infection were significantly more likely to have malnutrition compared to those with mild and moderate infection (OR: 5.3 [4–7.2]). Patients with comorbidities had a significantly higher risk of GLIM malnutrition than patients without comorbidities (OR: 2.4 [1.8–3.1]), but this increase in risk was not significant for malnutrition assessed by BMI. Patients who could not eat on their own had a higher risk of malnutrition according to both nutritional assessment tools. There was no significance in risk for malnutrition per BMI between enteral nutrition (EN) and total parenteral nutrition (TPN). 

Table 4 shows the associations between laboratory markers and malnutrition status as assess by GLIM and BMI. For malnutrition assessed by GLIM, hypoalbuminemia and hyponatremia were associated with increased odds of malnutrition (OR: 2.8 [1.7–4.5] and OR: 1.3 [1–1.7], respectively). 

For malnutrition assessed by BMI, we observed no significant associations between the laboratory markers and malnutrition status.

### 3.3. Refeeding Syndrome among COVID-19 Patients

The overall prevalence of risk of RS was 28.7%. The results described in Table 5 were statistically significant with *p* < 0.05. For the group of patients at risk of RS, the proportion of severe and critical patients at risk of RS was much higher than that of mild and moderate disease with 54.8% and 18.7%. Severe and critical COVID-19 increased the odds of risk for RS by 5.3 times, compared to mild and moderate. Severe and critical COVID-19 increased the risk of the incidence of RS by 2.47 times.

## 4. Discussion

Our study found that the proportion of malnutrition, the prevalence of patients at-risk of RS and the incidence of RS in COVID-19 patients were high, and could be related to the severity of COVID-19, number of comorbidities, and some laboratory tests.

Geriatric patients (defined as 65 years old or older) are 1.1 times more likely to be malnourished compared to those under 65 years of age; however, this difference is not statistically significant. Serious complications and deaths are in the geriatric population since older patients are at a higher risk for chronic diseases and co-morbidities such as diabetes mellitus, hypertension, COPD, and cancer amongst others compared to their younger counterparts [7]. Research by Recinella et al. reported that hospitalized elderly patients are at a higher nutrition-related risk, leading to higher mortality rate especially when combined with the two factors of low BMI and hypoalbuminemia [8]. Another paper authored by Pironi et al. in Italy, which used the GLIM nutritional assessment tool on 268 patients, showed that 50% of the participants had malnutrition and 70% of those malnourished patients are over 65 years of age [9]. This is supported by research on the nutritional status of COVID-19 patients who are 65 years and older in Wuhan, China using the Mini Nutritional Assessment (MNA) tool, which showed that the proportion of malnutrition among the geriatric patients was high at 52.7%, while 27.5% of patients was considered as at risk of malnutrition [10].

The overall malnutrition rate assessed by GLIM in this study was high. Of these, the elderly, those with severe or critical COVID-19, and those with comorbidities were more likely to have malnutrition. An observational longitudinal study with sample size of 160 patients found that the prevalence of malnutrition among COVID-19 patients was 42.1%, however the study found no significant difference in malnutrition status by age and presence of comorbidities [4].

Even though there is no consensus regarding the pathogenesis and clinical significance of hypoalbuminemia in medical literature, hypoalbuminemia is still considered to be associated with inflammation. This is due to the increase in capillary permeability, which allows serum albumin to escape and subsequently causes the interstitial space to expand and the distribution volume of albumin to rise [11]. Our study reveals that the average serum albumin level was significantly lower in those with more severe COVID-19 infection, and that hypoalbuminemia increased the odds of having malnutrition as assessed by GLIM. Hypoalbuminemia was related to several clinical outcomes of inpatients. In fact, the presence of hypoalbuminemia of approximately 10g/L is correlated with an increased risk of ICU admission, with the *p* value of less than 0.01 [4]. Pre-albumin is a shorter biologic half-life compared to albumin, and it may reflect current nutritional status of patients. However, similar to albumin, pre-albumin is also a hepatic protein and is affected by liver function, renal function and especially inflammatory state. According to a research paper by Jun Duan and associates on 348 mild COVID-19 cases at Chongqing Hospital, China [12], prealbumin levels of 15g/L at admission and lower increased the risk of severe disease progression by 6.6 when compared with those whose prealbumin was higher than 15 g/L.

Patients with COVID-19 experience multiple clinical conditions that may cause electrolyte imbalances. In this study, we found that the decline of electrolytes such as sodium, potassium and phosphorus were associated with increased risk of malnutrition, regardless of stastistical significance. Impaired renal function leads to fluid and electrolyte disturbances [13]. GI disorders can also lead to the aforementioned imbalances (the most common type is hypokalemia). We did not evaluate whether the electrolytes abnormalities may impact the poor outcome of patients; however, a study by Weili Wang proved this [14]. Fluid and electrolyte imbalances in COVID-19 patients are due to the reduction in the production of aldosterone, whose pathway is inhibited by medications. The aldosterone receptor, called mineralocorticoid receptor (MR), when activated, causes changes of ion concentrations—notably sodium and potassium—in order to maintain the physiological fluid and electrolytes balances [15]. MR is also expressed in a wide variety of organ tissues, such as the kidneys, the colon of the gastrointestinal tract, the central nervous system and the heart. Its expression in the large intestine means that the absorption and secretion of ions in the colon are disrupted when the aldosterone pathway is abnormal, which in turn triggers the imbalances mentioned above.

Normally, patients diagnosed with mild and moderate COVID-19 can eat and drink on their own. Patients with severe and critical levels may require invasive oxygen therapy support such as trachea intubation using a nasogastric tube or a nasojejunal tube. In our COVID-19 hospital, patients who are nil per os (NPO) due to the COVID symptoms or due to the need of oxygen therapy were at high risk of starvation and undernourishment. Other forms of nutrition such as enteral nutrition and parenteral nutrition were given to minimize this risk. We did not find any patients with normal nutritional status needing total parenteral nutrition. This explains that most well-nourished patients could consume orally or EN, sometimes if the calorie intake was <70% compared to the estimated energy requirement. PN was added to maintain adequate nutrition for patients. 

In terms of refeeding syndrome (RS), patients with severe or critical COVID-19 were significantly more likely than those with mild and moderate disease to have RS or be at risk of RS. In a malnourished state, there was a marked reduction of the storage of electrolytes, energy, and vitamins. When nutritional calories are reintroduced, a number of metabolic responses may occur, along with depleted intracellular stores and a large concentration gradient ensures a rapid fall in the extracellular concentration of ions such as potassium and phosphorus. If patients at risk of RS were not early evaluated and prevented and were started on nourishment, then potassium, phosphorus, and thiamin levels would decrease. If sustained for a long duration, these lowered levels of potassium, phosphorus, and thiamin lead to organ dysfunction such as arrythmia, respiratory failure, or even death. RS is a serious nutrition-related complication that deserves proper interest in clinical practice today. A prospective cohort study published in Frontiers in Nutrition [16] found that 82% of 327 critically ill COVID-19 patients were at risk of refeeding syndrome and 116 (36%) got involved in this syndrome. This result was higher than our study, as the cohort study only focused on critically ill patients, who could be at high risk of malnutrition and RS.

One of the limitations of our study was that we did not assess the alterations of markers regarding nutritional status such as vitamin B1, vitamin C, zinc and magnesium due to the limitation of laboratory equipment. In terms of 25-OH vitamin D, the number of patients who were indicated by physicians for this laboratory test was low. This is because doctors tend to be primarily concerned about changes in common markers such as electrolytes, albumin levels, blood-clotting markers and arterial blood gas tests.

## 5. Conclusions

The prevalence of malnutrition as assessed by GLIM was high in our study. Patients with severe and critical levels of Covid-19 condition; older ages; and with comorbidities were significantly more likely to have malnutrition compared to their counterparts. These aforementioned factors are also independent risk factors for malnutrition, along with the inability of eating independently, hypoalbuminemia and hyponatremia.

## Figures and Tables

**Table 1:** Demographic characteristics of the study participants.

Criteria		Quantity (n)	Percentage (%)
Gender	Male	603	49.9
	Female	604	50.1
Age	58.9 ± 20.7 (18–103)		
Weight (kg)	57.8 ± 11.3 (30–130)		
Height (cm)	160 ± 8		
BMI (kg/m^2^)	22.3 ± 3.3		
Comorbidities	None	344	28.5
	One–Two background diseases	586	48.6
	More than three ones	277	22.9
Level of COVID-19 condition	Mild	636	52.7
	Moderate	235	19.5
	Severe	132	10.9
	Critical	204	16.9
Oxygen therapy	None	749	62.1
	Mask	200	16.6
	Nasal prong	118	9.8
	HFNC	19	1.6
	NIV (CIPAP or BIPAP)	15	1,2
	Mechanical ventilation/ ECMO	106	8.8

**Table 2:** Values of laboratory tests classified by severity of COVID-19.

Laboratory Tests	Mean Value According to Severity of COVID-19				
	Mild (n = 636)	Moderate (n = 235)	Severe (n = 132)	Critical (n = 204)	*p* Value
Albumin (g/L) (n = 424)	35.6 ± 7 [18.1–49.3] (n = 105)	32.2 ± 5.1 [17.6–42.5] (n = 86)	30.9 ± 5 [14.2–42.4] (n = 73)	28.5 ± 4.5 [17.5–39.8] (n = 160)	*p* < 0.05 *
Pre-albumin (mg/dL) (n = 29)	15 ± 5.7 [8–24] (n = 6)	15 (n = 1)	13.5 ± 4.9 [8–20] (n = 4)	11.9 ± 5.2 [3–19] (n = 18)	*p* > 0.05 **
Na (mmol/L) (n = 1033)	135.6 ± 4.6 [107–155] (n = 504)	132.9 ± 5 [115–145] (n = 212)	134.4 ± 5.5 [110–157] (n = 125)	134.7 ± 7.6 [106–163] (n = 192)	*p* < 0.05 **
K (mmol/L) (n = 1033)	3.8 ± 0.4 [2.2–5.5] (n = 504)	3.9 ± 0.5 [2.8–5.4] (n = 212)	4 ± 0.7 [2.2–6.2] (n = 125)	4.1 ± 0.7 [2.3–6.7] (n = 192)	*p* < 0.05 **
*p* (mmol/L) (n = 45)	0	1 ± 0.2 [0.9–1.2] (n = 4)	1.2 ± 0.5 [0.6–1.8] (n = 4)	1.1 ± 0.4 [0.4–2.3] (n = 37)	*p* > 0.05 *
Triglyceride (mmol/L) (n = 22)	1.6 ± 0.9 [0.3–2.9] (n = 6)	2.2 ± 2.2 [0.6–6.1] (n = 5)	1.8 ± 1.1 [0.8–3.4] (n = 4)	3.9 ± 3 [1.1–11.7] (n = 10)	*p* > 0.05 **

* ANOVA test; ** Kruskal-Wallis test

**Table 3:** Association between nutritional status and age, gender, severity, comorbidities, feeding assistance and feeding route.

	Malnutrition According to GLIM				Malnutrition According to BMI			
	Normal n (%)	Malnutrition n (%)	OR [95% CI]	*p* Value	Normal n (%)	Malnutrition n (%)	OR [95% CI]	*p* Value
*Age*								
<65 (n = 663)	441 (36.5)	222 (18.4)	5.1 (4–6.6)	*p* < 0.05	617 (51.1)	46 (3.8)	1.6 (1–2.4)	*p* < 0.05
≥65 (n = 544)	152 (12.6)	392 (32.5)			487 (40.3)	57 (4.7)		
*Gender*								
Male (n = 603)	306 (25.4)	297 (24.6)	1.1 (0.9–1.4)	*p* > 0.05	558 (46.2)	45 (3.7)	1.3 (0.9–2)	*p* > 0.05
Female (n = 604)	287 (23.8)	317 (26.3)			546 (45.2)	58 (4.8)		
*Severity*								
Mild and moderate (n = 871)	520 (59.7)	351 (40.3)	5.3 (4–7.2)	*p* < 0.05	807 (67)	64 (5.3)	1.7 (1.1–2.6)	*p* < 0.05
Severe and critical (n = 336)	73 (21.7)	263 (78.3)			297 (24.6)	39(3.2)		
*Comorbidities*								
No (n = 344)	221 (66.1)	123 (33.9)	2.4 (1.8–3.1)	*p* < 0.05	316 (26.2)	28 (2.3)	1.1 (0.7–1.8)	*p* > 0.05
Yes (n = 863)	372 (43.1)	491 (56.9)			788 (65.3)	75 (6.2)		
*Feeding assistance*								
No (n = 856)	535 (44.3)	321 (2.7)	8.4 (6.1– 11.7)	*p* < 0.05	798 (66.1)	58 (4.8)	2 (1.3–3.1)	*p* < 0.05
Yes (n = 351)	58 (4.8)	293 (24.3)			306 (25.4)	45 (3.7)		
*Feeding route*								
EN and/or PN (n = 1196)	593 (49.1)	603 (50)		*p* < 0.05	1095 (90.7)	101 (8.4)	2.4 (0.2–11.9)	*p* > 0.05
TPN (n = 11)	0	11 (0.9)			9 (0.7)	2 (0.2)		

EN: Enteral nutrition; (T)PN: (Total) parenteral nutrition; OR: Odds Ratio.

**Table 4:** Association between laboratory tests and malnutrition according to GLIM and BMI.

	Malnutrition According to GLIM				Malnutrition According to BMI			
	Normal n (%)	Malnutrition n (%)	OR [95% CI]	*p* Value	Normal n (%)	Malnutrition n (%)	OR [95% CI]	*p* Value
Albumin								
Normal (n = 109)	49 (11.6)	60 (14.1)	2.8 (1.7–4.5)	*p* < 0.05	104 (24)	7 (1.7)	2.2 (1–6.1)	*p* > 0.05
Hypoalbuminemia (n = 315)	72 (17)	243 (57.3)			273 (64.4)	42 (9.9)		
Pre-albumin								
Normal (n = 2)	1 (3.4)	1 (3.4)	2.9 (0–235.2)	*p* > 0.05	2 (6.9)	0		*p* > 0.05
Low level (n = 27)	7 (24.2)	20 (69)			24 (82.8)	3 (10.3)		
Sodium								
Normal (n = 505)	259 (25)	246 (23.8)	1.3 (1–1.7)	*p* < 0.05	462 (44.7)	43 (4.2)	1 (0.7–1.7)	*p* > 0.05
Hyponatremia (n = 528)	232 (22.5)	296 (28.7)			481 (46.6)	47 (4.5)		
Potassium								
Normal (n = 933)	444 (43)	489 (47.3)	1 (0.7–1.6)	*p* > 0.05	850 (82.3)	83 (8)	0.8 (0.3–1.7)	*p* > 0.05
Hypokalemia (n = 100)	47 (4.6)	53 (5.1)			93 (9)	7 (0.7)		
Phosphorus								
Normal (n = 32)	7 (15.6)	25 (55.6)	3.4 (0.4–163.8)	*p* > 0.05	29 (64.4)	3 (6.8)	1.8 (0.1–17.3)	*p* > 0.05
Hypophosphatemia (n = 13)	1 (2.2)	12 (26.6)			11 (24.4)	2 (4.4)		

**Table 5:** The proportion of RS at-risk patients and RS according to severity of COVID-19 and the association between RS and severity of the disease.

Severity of COVID-19 (n = 1207)	At Risk of RS (Baseline)			Incidence of RS		
	n (%)	OR [95% CI]	*p* Value	n (%)	RR [95% CI]	*p* Value
Mild and moderate	163 (18.7)	1	<0.05	13 (1.5)	1	<0.05
Severe and critical	184 (54.8)	5.3 [4–6.9]		25 (7.4)	2.47 [1.93–3.17]	

## Data Availability

The data presented in this study are not publicly available due to confidentiality reasons. These data are available on request from the leading author, email: linhngthuy@hmu.edu.vn.
